# B-cell Acute Lymphoblastic Leukemia Presenting as Acute Liver Injury: A Case Report

**DOI:** 10.7759/cureus.79921

**Published:** 2025-03-02

**Authors:** Tsering Dolkar, Lakshmi Naidu, Eun Lee, Reinhold Munker

**Affiliations:** 1 Internal Medicine, University of Kentucky, Lexington, USA; 2 Pathology, University of Kentucky, Lexington, USA; 3 Hematology and Oncology, University of Kentucky HealthCare, Lexington, USA

**Keywords:** acute liver injury, b-cell acute lymphoblastic leukemia, blinatumomab, cytotoxic chemotherapy, hemophagocytic lymphohistiocytosis (hlh)

## Abstract

We present a case of a patient with jaundice who was referred to our facility due to markedly elevated liver enzymes and pancytopenia. The patient’s only symptom was jaundice, prompting an initial evaluation at an outside hospital, where laboratory tests revealed significantly elevated liver enzymes and pancytopenia, leading to referral for further assessment. The presence of both elevated liver enzymes and pancytopenia helped narrow the differential diagnoses. Persistent pancytopenia necessitated a bone marrow biopsy for definitive diagnosis. Additionally, given the ongoing elevation of liver enzymes without an apparent infectious or inflammatory cause, a liver biopsy was also performed. The results revealed leukemic infiltration of the liver and the presence of B-cell leukemia in the bone marrow. Further laboratory findings met the diagnostic criteria for hemophagocytic lymphohistiocytosis (HLH) secondary to leukemia. The patient was treated with steroid therapy for HLH, followed by chemotherapy and immunotherapy for B-cell acute lymphoblastic leukemia, leading to sustained remission and normalization of liver function. Thirteen months after diagnosis, the patient remains in good health and continues blinatumomab-prednisone, vincristine, 6-mercaptopurine, and methotrexate consolidation therapy.

## Introduction

Acute liver injury (ALI) is defined as coagulopathy with an international normalized ratio (INR) >2 or an INR >1.5 accompanied by any degree of hepatic encephalopathy, lasting less than 24 weeks [1]. The latter condition is also referred to as acute liver failure. ALI has a wide range of potential causes, making it essential to consider a broad differential when laboratory findings indicate liver injury. Common etiologies, such as viral hepatitis, drug-induced liver injury, and alcohol-related liver injury, should be systematically ruled out. A thorough patient history and additional laboratory parameters often help narrow the differential diagnosis.

Our patient was referred from another facility with pancytopenia and markedly elevated liver enzymes. Further evaluation revealed that the patient met the diagnostic criteria for hemophagocytic lymphohistiocytosis (HLH) [2]. Liver and bone marrow biopsies confirmed a diagnosis of B-cell acute lymphoblastic leukemia (B-ALL). Given the patient’s elevated liver enzymes, treatment was initiated with high-dose steroids for HLH, followed by a mini-dose chemotherapy regimen for B-ALL induction therapy, as full-dose chemotherapy was deemed unsuitable. A subsequent interval bone marrow biopsy showed no residual leukemic cells, and the patient proceeded with consolidation therapy using immunotherapy to allow further liver recovery.

This case underscores the importance of timely diagnosis and appropriate treatment in patients presenting with significant liver injury and pancytopenia secondary to leukemia.

## Case presentation

A male in his mid-20s with no significant medical history aside from herbal supplement use was referred from a regional hospital for pancytopenia and impending acute hepatic failure. On presentation, he was vitally stable, alert, and oriented to time, place, and person. His initial laboratory results showed alanine transaminase (ALT) and aspartate transaminase (AST) levels exceeding 7,000 IU/L, total bilirubin at 12.4 mg/dL, ALP at 357 IU/L, and an INR of 2.1 (Table 1).

**Table 1:** Liver function test results at different time intervals ALT, alanine transaminase; AST, aspartate transaminase; INR, international normalized ratio

Marker	Reference range	On admission	Week 4 (discharged)	Week 6 (one dose of blinatumomab)	Week 12 (full dose cytarabine)
ALT	10-50 U/L	>7,000	247	241	85
AST	10-50 U/L	>7,000	101	99	53
Total bilirubin	0.2-1.1 mg/dL	12.4	7.8	3	0.5
INR	0.9-1.1	2.1	1.1	1	1

The patient had no history of chronic liver disease or hepatitis and denied any over-the-counter medication use aside from herbal supplements. Physical examination was unremarkable except for icterus, and he remained hemodynamically stable apart from a fever of 100.6°F. An acute hepatitis panel was negative, and blood tests ruled out alcohol and acetaminophen toxicity. Liver function tests were subsequently monitored at different time intervals (Table 1).

His CBC revealed worsening pancytopenia (Table 2). A peripheral blood smear showed anemia with polychromasia and rare teardrop cells, along with leukopenia characterized by reactive and immature lymphocytes and rare blasts. His ferritin level was >100,000, and soluble IL-2 receptor (sIL2R) was 29,900. An ultrasound of the upper right quadrant was unremarkable.

**Table 2:** CBC on admission revealing pancytopenia ANC, absolute neutrophil count; Hb, hemoglobin; Plt, platelets

Marker	Value	Normal range
Hb	11.8	13.5-17.5 g/dL
Total WBC	1.6	3.7-10.3 × 10³/µL
ANC	0.7	1.6-6.1 × 10³/µL
Plt	79	155-369 × 10³/µL

Blood cultures were positive for methicillin-resistant *Staphylococcus epidermidis* bacteremia, secondary to left arm cellulitis. With ferritin >100,000, elevated triglycerides (TG), elevated sIL2R, pancytopenia, splenomegaly, and fever, the patient met six of the eight diagnostic criteria for HLH, as outlined in the HLH-2004 guidelines [2].

**Figure 1:**
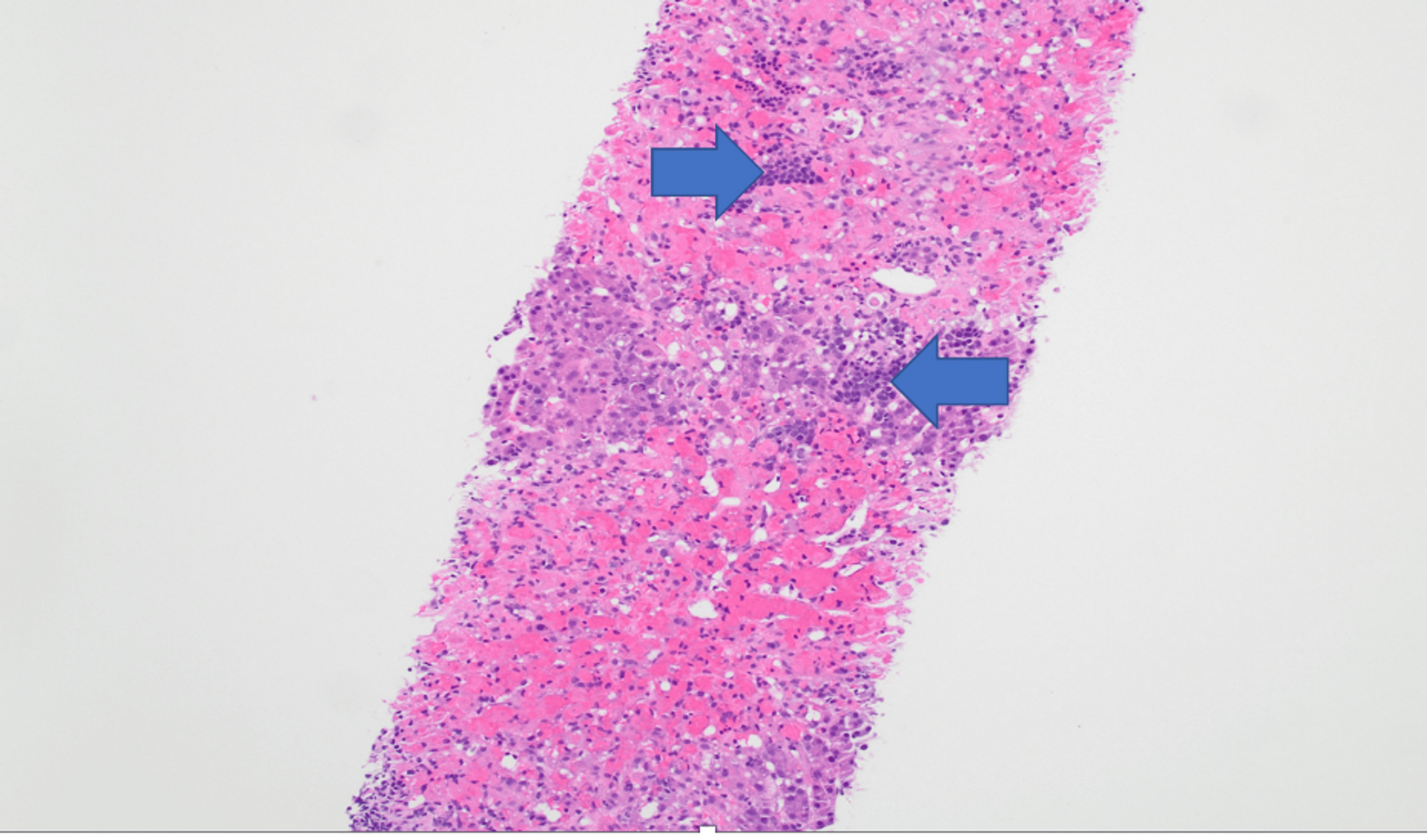
Liver biopsy revealing atypical cells (arrows) within the portal area (H&E 100×)

A bone marrow biopsy was performed, revealing evidence of B-ALL. The biopsy confirmed the presence of Philadelphia chromosome-negative B-lymphoblastic leukemia (Figure 1).

**Figure 2:**
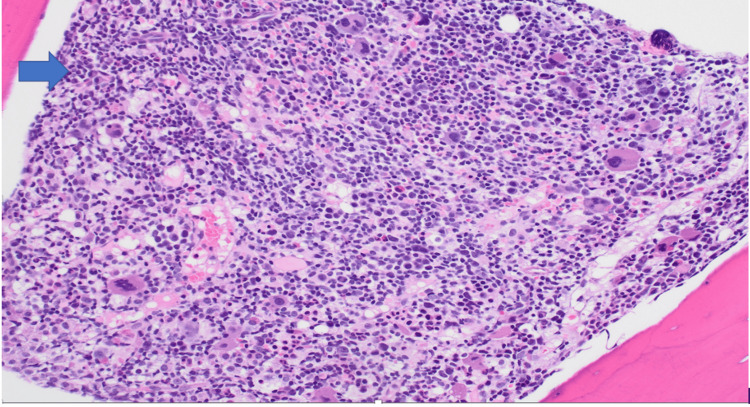
Bone marrow biopsy showing hypercellular bone marrow with B-lymphoblastic leukemia blasts (arrow) (H&E 100×)

Cytogenetic analysis was normal, while fluorescence in situ hybridization revealed a deletion of *CDKN2A* (9p21) and monosomy for chromosomes 9 and 17. A liver biopsy (Figure 2) demonstrated extensive centrilobular necrosis with minimal infiltration by leukemic cells. A scrotal ultrasound was unremarkable.

## Discussion

The liver plays a crucial role in detoxification and various metabolic processes. A pattern of elevated ALT/AST disproportionate to ALP and bilirubin typically indicates hepatocellular injury, whereas elevated ALP and bilirubin with relatively lower AST/ALT suggest a cholestatic injury pattern [3]. Isolated hyperbilirubinemia is characterized by increased bilirubin levels with normal alkaline phosphatase and AST/ALT levels. The R ratio helps classify liver injury as hepatocellular, cholestatic, or mixed. It is calculated using the formula R = (ALT value ÷ ALT upper limit of normal (ULN)) ÷ (ALP value ÷ ALP ULN). An R ratio above 5 suggests hepatocellular injury, a value below 2 indicates cholestatic injury and a ratio between 2 and 5 represents a mixed pattern [3].

Our patient had an R ratio of 7, indicative of hepatocellular injury. Ultrasound findings ruled out biliary obstruction, further supporting this diagnosis. Additionally, the patient exhibited elevated ferritin (>100,000), increased TG, elevated sIL2R, pancytopenia, splenomegaly, and fever - meeting the diagnostic criteria for HLH [2]. HLH is a rare but potentially fatal disorder characterized by the activation of cytotoxic T lymphocytes, natural killer cells, and macrophages, leading to hypercytokinemia and immune-mediated organ damage [2,4].

HLH can be genetic (primary) or acquired. Both forms involve impaired function of natural killer cells and cytotoxic T cells. The primary form results from genetic mutations affecting proteins critical for T and natural killer cell function, while the acquired form is typically triggered by infections - most commonly herpes viruses - as well as autoimmune conditions, immune deficiencies, and malignancies (particularly lymphomas) [2,4]. Persistent immune activation leads to hypercytokinemia, driving the clinical features of HLH. Initially, HLH diagnosis was based on five criteria: fever, splenomegaly, bicytopenia, hypertriglyceridemia and/or hypofibrinogenemia, and hemophagocytosis. In 2004, the Histiocyte Society expanded the criteria to include low or absent natural killer cell activity, hyperferritinemia, and elevated sIL2R (sCD25) levels. A diagnosis of HLH requires fulfillment of at least five of these eight criteria [2,4,5].

HLH can occur secondary to malignancies, including leukemia [6,7]. Reports describe cases where leukemia complicated by HLH led to acute liver failure [8,9]. Anderson et al. documented three leukemia patients presenting with acute liver failure, all of whom had poor prognoses and succumbed to the condition [10].

A liver biopsy in our patient revealed extensive centrilobular necrosis, cholestasis, lymphocytic infiltration (including leukemic cells), and extramedullary hematopoiesis (numerous hematopoietic cells, including megakaryocytes, secondary to splenomegaly). These findings suggested two concurrent processes: severe centrilobular necrosis responsible for the patient’s ALT elevation (>7,000) and possible leukemic infiltration. Given the patient’s clinical scenario - including sepsis, antibiotic treatment, and the use of herbal supplements (Herbalife QCarbo and Echinacea root) for six months - potential causes of necrosis included sepsis, hepatotoxicity from supplements, or a combination of both. However, the biopsy showed no histologic evidence of HLH. Common causes of extensive centrilobular necrosis include hypotension (“shock liver”), congestive heart failure, medications (including herbal supplements), sepsis, sinusoidal obstruction syndrome (veno-occlusive disease), and hepatic vein thrombosis (Budd-Chiari syndrome) [11].

The patient was started on high-dose dexamethasone (40 mg daily) for HLH, which was tapered over six weeks. A pediatric hematologist was consulted regarding fertility preservation in young adults. Following sperm collection, the patient was cleared for treatment initiation. Due to severe liver dysfunction, treatment began with mini-hyperfractionated cyclophosphamide, vincristine, doxorubicin, and dexamethasone (hyper-CVAD) [12]. Over four weeks, liver function improved, and the patient was discharged for outpatient follow-up.

One month after starting chemotherapy, repeat bone marrow and liver biopsies showed no morphological evidence of lymphoid blasts. Flow cytometry identified a minimal (0.3%) population of precursor B-cells, while minimal residual disease (MRD) testing was negative. Liver biopsy findings were consistent with near-resolution of centrilobular necrosis, with no evidence of leukemia involvement.

Liver enzyme levels continued to improve over subsequent weeks. Given the lack of morphologic evidence of leukemia, the patient was re-admitted for consolidation therapy with one cycle of blinatumomab. This decision was influenced by bilirubin levels remaining above 2, necessitating further liver recovery before resuming cytotoxic chemotherapy per hyper-CVAD with sequential blinatumomab and rituximab, as described in Jabbour et al.’s study [13].

Following blinatumomab, bone marrow biopsy revealed slightly hypocellular marrow with trilineage hematopoiesis and no morphological evidence of acute lymphoblastic leukemia (ALL). Flow cytometry was equivocal, and MRD remained negative. Bilirubin normalized, and ALT/AST/ALP levels, although still elevated, were less than three times the ULN. Thirteen months post-diagnosis, the patient remains well and is undergoing maintenance consolidation with blinatumomab-prednisone, vincristine, 6-mercaptopurine, and methotrexate (POMP) [13].

A review of extramedullary involvement in ALL identified 12 publications reporting liver involvement, with patients aged 4-18 years [14]. More recent case reports describe young patients with similar presentations [15,16]. As in our case, transaminitis may not be due to massive liver infiltration but rather to lymphoblast adhesion to hepatic portal veins and venules, triggering a toxic immune response. Limited follow-up data make the prognosis unclear, but it may be comparable to other patients with ALL of the same age group.

Leukemic cells use adhesion molecules to navigate and colonize various anatomical sites, including lymph nodes, spleen, bone marrow, and, less commonly, the liver. These molecules mediate interactions with vascular endothelium and extracellular matrix components, influencing cell trafficking and localization [17]. While the liver contains macrophages expressing sialoadhesin (SIGLEC-1), which binds sialic acids on leukocytes, it has lower expression of adhesion molecules facilitating leukemic cell homing compared to lymphoid organs such as the lymph nodes and spleen [18]. This could explain the relatively low incidence of leukemic infiltration in the liver, although further research is warranted.

## Conclusions

Given the continued recovery of his liver function, the patient underwent consolidation cycles with hyper-CVAD, followed by sequential blinatumomab and rituximab, and then maintenance therapy with blinatumomab-POMP. He remains in remission and continues regular follow-ups with his hematologist.

This case underscores the importance of recognizing ALI as a potential manifestation of hematologic malignancy. In such instances, a bone marrow biopsy should be performed without delay. Even when HLH is strongly suspected, it is crucial not to overlook an underlying malignancy. This is especially relevant in younger patients, where liver involvement may not be uncommon but often remains asymptomatic, as seen in this case.
